# Isaacs syndrome with LGI1 and CASPR2 antibodies after HPV vaccination: A case report

**DOI:** 10.1097/MD.0000000000035865

**Published:** 2023-11-03

**Authors:** Bufan Yang, Wei Wei, Jingfeng Duan, Pei Xiao, Yu Jing, Yufeng Tang

**Affiliations:** a Department of Neurology, Mianyang Central Hospital, School of Medicine, University of Electronic Science and Technology of China, Mianyang, Sichuan, People’s Republic of China; b Department of Neurology, University Medical Center of Göttingen, Georg-August-University of Göttingen, Göttingen, Lower Saxony, Germany.

**Keywords:** anti-sulfatide antibody, CASPR2, HPV, Isaacs syndrome, LGI1

## Abstract

**Rationale:**

Isaacs syndrome is peripheral nerve hyperexcitability characterized by spontaneous muscle twitching and rigidity and is often associated with antibodies to CASPR2 (contactin-associated protein-like 2) and LGI1 (leucine-rich glioma-inactivated 1). But it is a rare Isaacs syndrome with LGI1 and CASPR2 antibodies after human papilloma virus (HPV) vaccination.

**Patient concerns:**

The patient presented with limb pain, muscle twitching, numbness in the extremities and around the mouth, and hand rash after the second dose of HPV vaccine.

**Diagnoses:**

Laboratory tests indicated positive for LGI1 antibodies, CASPR2 antibodies, anti-phosphatidylserine/prothrombin antibodies and anti-sulfatide antibodies, TPO and ATG, IgG E. The patient post-M-wave discharges were seen on F-wave examination of the posterior tibial nerve in both lower limbs. We diagnosis the patient with Isaacs syndrome.

**Interventions:**

Treatment with the intravenous immunoglobulin (IVIG) treatment, after 5 days of IVIG therapy (0.4 mg/kg/day), the rash on the hand disappeared, the pain was relieved, the sleep improved.

**Outcomes:**

After 3 Courses of treatment, the clinical manifestations of the nervous system disappeared and negative responsibility antibodies profile.

**Lessons:**

This case report suggests a possible adverse reaction to HPV vaccination, which could be treated by attempting several periods of IVIG therapy. The underlying immune mechanisms need to be studied with further extensive data.

## 1. Introduction

Isaacs syndrome, first described by Hyam Isaacs in 1961,^[1]^ is a syndrome of acquired autoimmune disease in which peripheral nerve hyperexcitability predominates, characterized by spontaneous twitching and rigidity of muscles, spasticity and autonomic disturbances, occasionally accompanied by neuropathic pain and paresthesia. Electromyography can reveal spontaneous irregular discharges of motor nerve fibers. Indeed, a significant proportion of Isaacs syndrome cases have detectable autoantibodies, and it is generally believed that the target antigens are contactin-associated protein-like 2 (CASPR2) and leucine-rich glioma-inactivated 1 (LGI1) of the presynaptic membrane of the neuromuscular junction.^[2,3]^ Patients may therefore benefit from plasma exchange or intravenous immunoglobulin (IVIG) therapy.^[2]^ The exact cause of Isaacs syndrome remains unclear and may be related to genetic, autoimmune and paraneoplastic factors.^[2–4]^ The quadrivalent human papillomavirus (qHPV) vaccine, first approved in 2006, is a highly effective prophylaxis against papillomavirus types 6, 11, 16, and 18. Since the vaccine was approved, studies have investigated the possibility of autoimmune disease following application of the vaccine.^[5–9]^ Here, we report the clinical features of a case of Isaacs syndrome that occurred after the second dose of qHPV vaccination and their response to symptomatic and immunomodulatory treatment, which was positive for CASPR2 antibodies and positive for LGI1 antibodies with positive antiphospholipid antibodies and positive anti-sulfatide antibodies, TPO and ATG, IgG E.

## 2. Case presentation

### 2.1. Basic information of patient

A 28-year-old female presented with pain in her left buttock and lower limb on the third day after her second dose of the quadrivalent human papilloma vaccine. The patient had received her first vaccination with the same dose and type 3 months before. The patient presented with left-sided buttocks and lower limb pain, which worsened with activity and got progressively worse during the pain, in the buttocks, arm and leg, with discontinuous involuntary muscle twitches, with limb numbness and perioral, with hand rash, poor appetite and poor sleep. There was no family history or disease in the past.

#### 2.1.1. Neurological examination.

**Figure 1.**
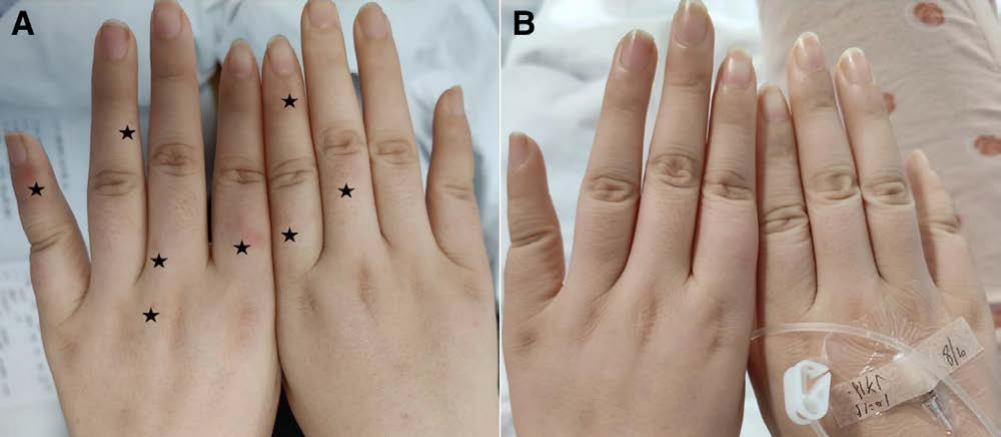
Hands scattered in rash (A); rash disappeared after treatment (B).

The patient vital signs, mental status, cranial nerves, muscle tone, and cerebellar signs were normal. Right upper limb strength level 5, right lower limb strength level 4, left upper limb strength level 5, left lower limb strength level 4. There was obvious limb pain in both lower limbs. It was considered that the muscle strength test results were affected by the patient limb pain. Scattered red rashes were seen on both hands (Fig. 1A): Touch, temperature, vibration, and proprioception exist symmetrically in the limbs, and the limbs are allergic to acupuncture pain. Bilateral biceps, triceps, radial membrane reflex, knee reflex (+), and bilateral ankle reflex disappeared. Pathological signs and meningeal irritation were negative. Numerical rating scale (NRS) was used to score the pain, and the pain score was 10. The pain and discomfort in the whole body, especially in the hip, the left lower limb, and the proximal extremities of both upper limbs, occurred in waves, lasted for a long time, and worsened after activities, accompanied by generalized muscle jumping pain in the whole body, numbness in limbs, mouth, and nose.

#### 2.1.2. Lab examination.

Immunoglobulin E 170IU/mL (<100); Thyroglobulin antibody-positive, anti-thyroid peroxidase antibody-positive, electrolytes: sodium 134 mmol/L (137–147 mmol/L) (Table 1). Other serum examinations were in the normal ranges (Table 2). Imagological examination: chest computerized tomography (CT) scan: not thymoma. Other examination including video electroencephalogram, routine electrocardiogram, simple cognitive scale, abdominal CT, cranial MRI (magnetic resonance imaging), thoracolumbar MRI, left thigh MRI and positron emission tomography-computed tomography were normal.

**Table 1:** Results of various immune-related antibodies tests.

Inspection antibodies	Abbreviation	Test date					Reference range	Units	Test method
		08/05.2021	09/06.2021	11/12.2021	03/07.2022	01/15.2023			
Anti-leucine-rich glioma Inactivated Protein 1 IgG antibody	LGI 1	Positive (+) 1:30	Positive (+) 1:10	Negative (−)	Negative (−)	N/A	Negative (−)	N/A	CBA
Anti-contact protein associated Protein 2 IgG antibody	CASPR2	Positive (+) 1:100	Positive (+) 1:100	Positive (+) 1:10	Negative (−)	N/A	Negative (−)	N/A	CBA
Anti-sulfatide IgG antibody	Sulf	Positive (+)	Positive (+)	Negative (−)	Negative (−)	N/A	Negative (−)	N/A	WB
Anti-GD2 IgM antibody	GD2	Negative (−)	Positive (+)	Positive (+)	Positive (+)	N/A	Negative (−)	N/A	WB
Anti-phosphatidylserine/prothrombin IgM antibody	aPS/PT	36.26	N/A	N/A	67.17	N/A	0–30	U/mL	ELISA
Anti-thyroglobulin antibody	ATG	Positive (+)	N/A	N/A	N/A	Negative (−)	Negative (−)	N/A	CL
Anti-thyroid peroxidase antibody	TPO	Positive (+)	N/A	N/A	N/A	Negative (−)	Negative (−)	N/A	CL
Immunoglobulin E	IgE	170	N/A	N/A	N/A	42	< 100	IU/mL	CL
Serum sodium ion	Na+	134	137.5	137.8	N/A	137.3	137–147	mmol/L	ISE

CASPR2 = contactin-associated protein-like 2, CBA = cell based fluorescence assay, CL = chemiluminescence assay, ELISA = enzyme-linked immunosorbent assay, ISE = ion selective electrode method, WB = western blotting analysis.

**Table 2:** Results of all tests.

Inspection antibody	Abbreviation	Test date					Reference range	Units	Test method
		08/05.2021	09/06.2021	11/12.2021	03/07.2022	01/15.2023			
Perinuclear Anti-neutrophil cytoplasmic antibody	pANCA	Negative (−)	N/A	N/A	N/A	N/A	Negative (−)	N/A	IF
Cytoplasmic Anti-neutrophil cytoplasmic antibody	cANCA	Negative (−)	N/A	N/A	N/A	N/A	Negative (−)	N/A	IF
Anti-protease 3 antibody	PR3-Ab	Negative (−)	N/A	N/A	N/A	N/A	Negative (−)	N/A	WB
Anti-myeloperoxidase antibody	MPO-Ab	Negative (−)	N/A	N/A	N/A	N/A	Negative (−)	N/A	WB
Anti-glomerular basement membrane antibody	GBM	Negative (−)	N/A	N/A	N/A	N/A	Negative (−)	N/A	ELISA
Immunoglobulin G4	G4	311.8	N/A	N/A	N/A	N/A	39.2–864.0	mg/L	IT
Anti-β2 glycoprotein 1IgA antibody	β2 GPI-IgA	3.75	N/A	N/A	< 2.00	N/A	< 20.00	RU/mL	CL
Anti-β2 glycoprotein 1IgM antibody	β2 GPI-IgM	5.29	N/A	N/A	2.61	N/A	< 20.00	RU/mL	CL
Anti-β2 glycoprotein 1IgG antibody	β2 GPI-IgG	< 2.00	N/A	N/A	< 2.00	N/A	< 20.00	RU/mlL	CL
Anti-cardiolipin IgG antibody	ACL-IgG	< 2.00	N/A	N/A	< 2.00	N/A	< 20.00	RU/mL	CL
Anticardiolipin IgA antibody	ACL-IgA	< 2.00	N/A	N/A	< 2.00	N/A	< 20.00	RU/mL	CL
Anti-cardiolipin IgM antibody	ACL-IgM	6.37	N/A	N/A	4.11	N/A	< 20.00	RU/mL	CL
Melanoma differentiation-associated gene 5 antibody	anti-MDA5	Negative (−)	N/A	N/A	N/A	N/A	Negative (−)	N/A	CBA
Anti-OJ antibody		Negative (−)	N/A	N/A	N/A	N/A	Negative (−)	N/A	WB
Anti-KS antibody		Negative (−)	N/A	N/A	N/A	N/A	Negative (−)	N/A	WB
Anti-ZO antibody		Negative (−)	N/A	N/A	N/A	N/A	Negative (−)	N/A	WB
Anti-HA antibody		Negative (−)	N/A	N/A	N/A	N/A	Negative (−)	N/A	WB
Anti-Scl-70 antibody		Negative (−)	N/A	N/A	N/A	N/A	Negative (−)	N/A	WB
Anti-PM-SCL100 antibody		Negative (−)	N/A	N/A	N/A	N/A	Negative (−)	N/A	WB
Anti-PM-SCL75 antibody		Negative (−)	N/A	N/A	N/A	N/A	Negative (−)	N/A	WB
Anti-KU antibody		Negative (−)	N/A	N/A	N/A	N/A	Negative (−)	N/A	WB
Anti-RNA-PIII antibody		Negative (−)	N/A	N/A	N/A	N/A	Negative (−)	N/A	WB
Anti-Th/To antibody		Negative (−)	N/A	N/A	N/A	N/A	Negative (−)	N/A	WB
Anti-Fibrillarin antibody		Negative (−)	N/A	N/A	N/A	N/A	Negative (−)	N/A	WB
Anti-NOR-90 antibody		Negative (−)	N/A	N/A	N/A	N/A	Negative (−)	N/A	WB
Anti-JO-1 antibody		Negative (−)	N/A	N/A	N/A	N/A	Negative (−)	N/A	WB
Anti-PL-7 antibody		Negative (−)	N/A	N/A	N/A	N/A	Negative (−)	N/A	WB
Anti-PL-12 antibody		Negative (−)	N/A	N/A	N/A	N/A	Negative (−)	N/A	WB
Anti-EJ antibody		Negative (−)	N/A	N/A	N/A	N/A	Negative (−)	N/A	WB
Anti-SRP antibody		Negative (−)	N/A	N/A	N/A	N/A	Negative (−)	N/A	WB
Anti-Mi-2 antibody		Negative (−)	N/A	N/A	N/A	N/A	Negative (−)	N/A	WB
Anti-TIF1γ antibody		Negative (−)	N/A	N/A	N/A	N/A	Negative (−)	N/A	WB
Anti-Ro-52 antibody		Negative (−)	N/A	N/A	N/A	N/A	Negative (−)	N/A	WB
Anti-SAE1 antibody		Negative (−)	N/A	N/A	N/A	N/A	Negative (−)	N/A	WB
Anti-SAE2 antibody		Negative (−)	N/A	N/A	N/A	N/A	Negative (−)	N/A	WB
Anti-NXP2 antibody		Negative (−)	N/A	N/A	N/A	N/A	Negative (−)	N/A	WB
Anti-phosphatidylserine/prothrombin IgG antibody	aPS/PT	3.37	N/A	N/A	8.29	N/A	0.00–30.00	U/mL	ELISA
Anti-double-stranded DNA antibody	dsDNA	Negative (−)	N/A	N/A	N/A	N/A	Negative (−)	N/A	ELISA
Acetylcholine receptor antibody	AchR.Ab	< 0.01	N/A	N/A	N/A	N/A	< 0.45	nmol/L	ELISA
Muscle-specific tyrosine kinase antibody	MuSK.Ab	0.25	N/A	N/A	N/A	N/A	< 0.40	U/mL	ELISA
Titin antibody	Titin-Ab	0.01	N/A	N/A	N/A	N/A	< 15.00	U/mL	ELISA
Ryanodine receptor antibody	RyR.Ab	Negative (−)	N/A	N/A	N/A	N/A	Negative (−)	N/A	ELISA
Anti-glutamate receptor (NMDA type) IgG antibody	Anti-NMDA	Negative (−)	Negative (−)	Negative (−)	Negative (−)	N/A	Negative (−)	N/A	CBA
Anti-glutamate Receptor AMPA1 IgG antibody	Anti-AMPA1	Negative (−)	Negative (−)	Negative (−)	Negative (−)	N/A	Negative (−)	N/A	CBA
Anti-glutamate receptor AMPA2 IgG antibody	Anti-AMPA2	Negative (−)	Negative (−)	Negative (−)	Negative (−)	N/A	Negative (−)	N/A	CBA
Anti-gamma-aminobutyric acid Type B Receptor IgG antibody	Anti-GABA B	Negative (−)	Negative (−)	Negative (−)	Negative (−)	N/A	Negative (−)	N/A	CBA
Anti-GM1 antibody IgG		Negative (−)	Negative (−)	Negative (−)	Negative (−)	N/A	Negative (−)	N/A	WB
Anti-GM2 antibody IgG		Negative (−)	Negative (−)	Negative (−)	Negative (−)	N/A	Negative (−)	N/A	WB
Anti-GM3 antibody IgG		Negative (−)	Negative (−)	Negative (−)	Negative (−)	N/A	Negative (−)	N/A	WB
Anti-GM4 antibody IgG		Negative (−)	Negative (−)	Negative (−)	Negative (−)	N/A	Negative (−)	N/A	WB
Anti-GD1a antibody IgG		Negative (−)	Negative (−)	Negative (−)	Negative (−)	N/A	Negative (−)	N/A	WB
Anti-GD1b antibody IgG		Negative (−)	Negative (−)	Negative (−)	Negative (−)	N/A	Negative (−)	N/A	WB
Anti-GD2 antibody IgG		Negative (−)	Negative (−)	Negative (−)	Negative (−)	N/A	Negative (−)	N/A	WB
Anti-GD3 antibody IgG		Negative (−)	Negative (−)	Negative (−)	Negative (−)	N/A	Negative (−)	N/A	WB
Anti-GT1a antibody IgG		Negative (−)	Negative (−)	Negative (−)	Negative (−)	N/A	Negative (−)	N/A	WB
Anti-GT1b antibody IgG		Negative (−)	Negative (−)	Negative (−)	Negative (−)	N/A	Negative (−)	N/A	WB
Anti-GQ1b antibody IgG		Negative (−)	Negative (−)	Negative (−)	Negative (−)	N/A	Negative (−)	N/A	WB
Anti-Sulfatide antibody IgM	Sulf	Negative (−)	Negative (−)	Negative (−)	Negative (−)	N/A	Negative (−)	N/A	WB
Anti-GM1 antibody IgM		Negative (−)	Negative (−)	Negative (−)	Negative (−)	N/A	Negative (−)	N/A	WB
Anti-GM2 antibody IgM		Negative (−)	Negative (−)	Negative (−)	Negative (−)	N/A	Negative (−)	N/A	WB
Anti-GM3 antibody IgM		Negative (−)	Negative (−)	Negative (−)	Negative (−)	N/A	Negative (−)	N/A	WB
Anti-GM4 antibody IgM		Negative (−)	Negative (−)	Negative (−)	Negative (−)	N/A	Negative (−)	N/A	WB
Anti-GD1a antibody IgM		Negative (−)	Negative (−)	Negative (−)	Negative (−)	N/A	Negative (−)	N/A	WB
Anti-GD1b antibody IgM		Negative (−)	Negative (−)	Negative (−)	Negative (−)	N/A	Negative (−)	N/A	WB
Anti-GD3 antibody IgM		Negative (−)	Negative (−)	Negative (−)	Negative (−)	N/A	Negative (−)	N/A	WB
Anti-GT1a antibody IgM		Negative (−)	Negative (−)	Negative (−)	Negative (−)	N/A	Negative (−)	N/A	WB
Anti-GT1b antibody IgM		Negative (−)	Negative (−)	Negative (−)	Negative (−)	N/A	Negative (−)	N/A	WB
Anti-GQ1b antibody IgM		Negative (−)	Negative (−)	Negative (−)	Negative (−)	N/A	Negative (−)	N/A	WB
Complement C3	C3	Negative (−)	N/A	N/A	N/A	Negative (−)	Negative (−)	N/A	CL
Complement C4	C4	Negative (−)	N/A	N/A	N/A	Negative (−)	Negative (−)	N/A	CL
Immunoglobulin A	IgA	Negative (−)	N/A	N/A	N/A	Negative (−)	Negative (−)	N/A	CL
Immunoglobulin G	IgG	Feminine (−)	N/A	N/A	N/A	Negative (−)	Negative (−)	N/A	CL
Immunoglobulin M	IgM	Negative (−)	N/A	N/A	N/A	Negative (−)	Negative (−)	N/A	CL
Anti- PM-SCL antibody	Anti-PM	Negative (−)	N/A	N/A	N/A	Negative (−)	Negative (−)	N/A	CL
Anti-polymyositis antibody	Anti-Jo1	Negative (−)	N/A	N/A	N/A	Negative (−)	Negative (−)	N/A	CL
Anti-sjogren Syndrome A	Anti-SSA	Negative (−)	N/A	N/A	N/A	Negative (−)	Negative (−)	N/A	CL
Anti-sjogren Syndrome B	Anti-SSB	Negative (−)	N/A	N/A	N/A	Negative (−)	Negative (−)	N/A	CL
Anti-sjogren Syndrome Ro52	Anti-Ro52	Negative (−)	N/A	N/A	N/A	Negative (−)	Negative (−)	N/A	CL
Antinuclear antibody	ANA-IIF	Negative (−)	N/A	N/A	N/A	Negative (−)	Negative (−)	N/A	IF
Anti-nuclear karyotype 1 titer	ANA-T1D	Negative (−)	N/A	N/A	N/A	Negative (−)	Negative (−)	N/A	CL
Anti-ribonucleoprotein antibody	Anti-RNP	Negative (−)	N/A	N/A	N/A	Negative (−)	Negative (−)	N/A	CL
Anti-ribosome P protein antibody	Anti-rib	Negative (−)	N/A	N/A	N/A	Negative (−)	Negative (−)	N/A	CL
Anti-nucleosome antibody	ANuA	Negative (−)	N/A	N/A	N/A	Negative (−)	Negative (−)	N/A	CL
Anti-acid nucleoprotein	Anti-Sm	Negative (−)	N/A	N/A	N/A	Negative (−)	Negative (−)	N/A	CL
Anti- double-stranded DNA	Anti-dSDNA	Negative (−)	N/A	N/A	N/A	Negative (−)	Negative (−)	N/A	CL
Anti-systemic sclerosis antibody	Anti-Sc1	Negative (−)	N/A	N/A	N/A	Negative (−)	Negative (−)	N/A	CL
Anti-cyclin antibody	Anti-PCNA	Negative (−)	N/A	N/A	N/A	Negative (−)	Negative (−)	N/A	CL
Anti-mitochondrial M2 antibody	AMA M2	Negative (−)	N/A	N/A	N/A	Negative (−)	Negative (−)	N/A	CL
Anti-centromere B antibody	Anti-cenPb	Negative (−)	N/A	N/A	N/A	Negative (−)	Negative (−)	N/A	CL
Anti-histone antibody	Anti-His	Negative (−)	N/A	N/A	N/A	Negative (−)	Negative (−)	N/A	CL
Rheumatoid factors	RF	Negative (−)	N/A	N/A	N/A	Negative (−)	Negative (−)	N/A	CL

CBA = cell based fluorescence assay, CL = chemiluminescence assay, ELISA = enzyme-linked immunosorbent assay, IF = immunofluorescence staining assay, ISE = ion selective electrode method, IT = immunoturbidimetry assay, WB = western blotting analysis.

**Figure 2.**
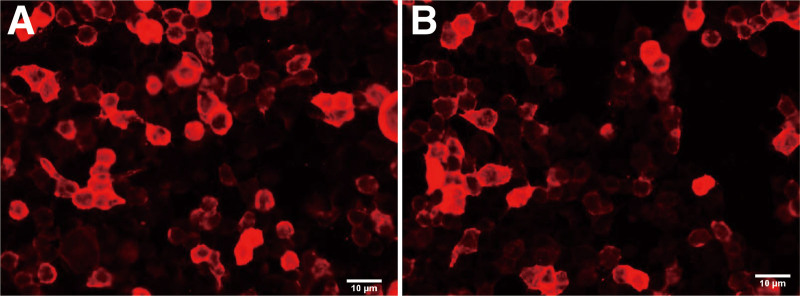
Patients antibodies reacted with Mammalian cells transfected with contactin-associated protein-like 2 (CASPR2) (A) and leucine-rich glioma-inactivated 1 (LGI1) (B). Diluted serum samples (1:10) were collected on August 5th, 2021. Reacted with mammalian cells with CASPR2 and LGI1 on August 7th, 2021. Scale bars, 10 μm (A) and (B).

**Figure 3.**
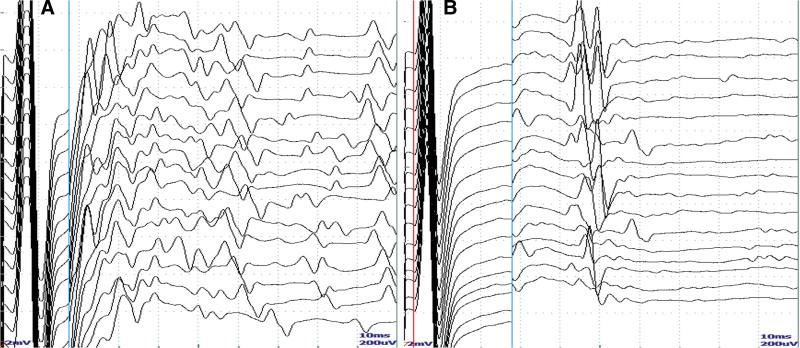
On the third day after admission, there was no bundle flutter or myoflutter potential in the patient EMG (The first EMG test was conducted on August 5th, and intravenous immunoglobulin (IVIg) treatment on August 6th), and post-M wave discharge was observed during F-wave examination of the posterior tibial nerve of both lower limbs (A); the discharge after F wave and M wave in posterior tibial nerves disappeared after treatment (B), the second EMG test was performed on the day 3 of hospitalization (admitted on September 26th, IVIg treatment on the same day, and examination on September 28th). The first test was conducted on the third day of hospitalization (admitted on August 3rd, examination on August 5th, and intravenous immunoglobulin (IVIg) treatment on August 6th). The Electromyography test was performed on the third day of hospitalization (admitted on September 26th, IVIg treatment on the same day, and examination on September 28th).

By the cell-based indirect immunofluorescence method (CBA, Sichuan kingmed center for clinical laboratory) to detect serum antibodies. The assay is based on the principle of transfecting an autoimmune encephalitis antigen gene into mammalian cells, in which the corresponding antigen is specifically expressed and green fluorescent protein is also expressed during transfection as an internal reference for detection. The results showed positive CASPR2 (1:100, results with a titer 1: <10 were considered negative) antibodies (Fig. 2A), and LGI1 (1:30, results with a titer 1: <10 were considered negative) (Fig. 2B), and Anti-Sulfatide IgG: positive, anti-phosphatidyl serine/prothrombin IgM antibody: 36.26U/mL (Table 1). Other serum antibodies were negative (Table 2). On the third day after admission (before IVIG therapy), there was no bundle flutter or myoflutter potential in the patient EMG, and post-M wave discharge was observed during F-wave examination of the posterior tibial nerve of both lower limbs (On the third day after admission, before the administration of intravenous immunoglobulin). The patient had no fasciculations or myofibrillation potentials on EMG, and post-M-wave discharges were seen on F-wave examination of the posterior tibial nerve in both lower limbs (Fig. 3A). After 5 days of IVIG therapy (0.4 mg/kg/day), the rash on the hand disappeared (Fig. 1B), the pain was relieved (pain score 3), the sleep improved, and the condition was improved and discharged.

#### 2.1.3. Outcomes of follow-up.

After discharge, gabapentin 0.3 g quaque nocte was continued. 46 days later, the patient was hospitalized again due to worsening pain (pain score 5). On neurological examination, the patient vital signs, mental status, cranial nerves, muscle strength, muscle tone, and cerebellar signs were normal. The sense of touch, temperature, acupuncture pain, vibration, and proprioception of limbs were normal and symmetrical. Bilateral biceps, triceps, radial membrane reflex (++), knee reflex (+), bilateral ankle reflex (++). Pathological signs and meningeal irritation were negative. NRS was used to score the pain, and the pain score was 5. Sore distension pain of extremities and buttocks appeared in waves; each attack lasted for a short time, worsened after activity, slightly relieved after rest, and was not accompanied by generalized muscle jumping pain of the whole body, numbness of limbs, mouth, and nose. No nerve muscle tonic and muscle fibrillation.

Laboratory examination, which showed positive CASPR2 (1:100, results with a titer 1: <10 were considered negative) antibodies, and LGI1 (1:10, results with a titer 1: <10 were considered negative), and Anti-Sulfatide IgG positive, Anti-gd2 IgM positive (Table 1). The patient was treated with IVIG (0.4 mg/kg/day) for 5 days. Electromyography was performed on day 3 of the second hospitalization (day 3 of IVIG therapy). Compared with the first electrophysiological examination, the conduction amplitude of sensory nerves in both upper limbs were significantly increased, and the discharge after F wave and M wave in both posterior tibial nerves disappeared (Fig. 3B), and the pain was relieved (pain score 2). She continued to take gabapentin 0.1g 3 times a day after discharge from the hospital. Two months later, the patient pain (pain score of 3) worsened again.

The neurological examination showed that the patient vital signs, mental status, cranial nerves, muscle strength, muscle tone, and cerebellar signs were all within normal limits. Sensory functions, including touch, temperature perception, acupuncture pain, vibration sense, and proprioception of the limbs, were normal and symmetrical. No abnormalities were observed in limb pain, touch sensation, vibration perception, or range of motion, which were all symmetrical. The bilateral biceps, triceps, and radial membrane reflexes were elicited and graded as (++), indicating normal responses. The knee reflexes were also graded as (++), bilaterally. Similarly, the ankle reflexes were graded as (++), bilaterally. No pathological signs or signs of meningeal irritation were detected. The pain experienced by the patient was assessed using the NRS, and the score was recorded as 3. The patient reported left lower limb and left hip distension pain that occurred in waves, with each episode being short-duration. The pain intensity worsened after physical activity but was slightly relieved after rest. No accompanying symptoms were reported. Enhanced CT of the chest showed no definite change from before. CASPR2 antibody 1:10, LGI1 antibody negative, Anti-Sulfatide IgG: negative, Anti-gd2 IgM positive (Table 1). After 5 days of treatment with IVIG (0.4 mg/kg/day), the pain was completely relieved. At the outpatient follow-up 4 months after his third discharge, CASPR2 antibody and LGI1 antibody negative, Anti-Sulfatide IgG: negative, Anti-gd2 IgM positive, anti- phosphatidylserine/prothrombin antibodies positive (Table 1). Fourteen months after discharge for the third time, the results of TPO, ATG, and IgG E were all negative (Table 1), and the patient had no clinical symptoms.

## 3. Discussion

CASPR2 and LGI1 are important components of voltage-gated Kv1 potassium channel complexes, widely expressed in the central and peripheral nervous systems. Autoimmune CASPR2 and LGI1 diseases usually manifest as Morvan syndrome and/or limbic encephalitis. We report a case of Isaacs with positive CASPR2, LGI1, anti-phospholipid antibody, and anti-Sulfatide antibody after the second dose of qHPV vaccine, and described the classic clinical feature of Isaacs: muscle spasms, which was confirmed by Cerami and Maryam Hatami.^[10,11]^ In fact, Isaacs syndrome is an autoimmune disease characterized by neurogenic myotonia. It is distinguished by the presence of a myotonic tremor syndrome resulting from heightened excitability of the peripheral nerves. Additionally, individuals with Isaac syndrome may experience pain and disruptions in autonomic function.^[4,12,13]^ It has been reported that in some cases, Isaacs syndrome in the central nervous system shows symptoms leading to hallucinations, dancing, insomnia, and intracranial hippocampal lesions.^[14]^ Although one study reported a case of CASPR2 and LGI1 double antibody positivity, the clinical presentation was GBS-like syndrome that developed into typical respiratory paralysis, and the neurological symptoms in this patient resolved quickly after plasma washing.^[15,16]^ The patient we reported presented with a unique combination of positive antibodies, including CASPR2, LGI1, antiphospholipid, and anti-Sulfatide antibodies. Notably, during the physical examination, we observed a weakened tendon reflex, a rare symptom resembling Guillain-Barre syndrome. However, the specific clinical significance of this Guillain-Barre-like symptom in relation to Isaac syndrome remains uncertain. Our patient also had intractable insomnia and antibody positivity, but no intracranial lesions were found. Studies have found that patients with both anti-CASPR2 and anti-LGI1 antibodies are at risk of thymoma.^[17,18]^ In some tumor-associated syndromes, neurological deficits may occur before the tumor is detected, so patients with negative malignancy should be followed for a long time. In this case, although screening for systemic malignancy was negative, the patient should be followed up for a long time. Concerning the treatment of this disease, studies have found inconsistent efficacy of IVIG or plasma exchange in this syndrome, mainly suggesting that high-dose steroid therapy should be tried when the IVIG (0.4 mg/kg/day, over 5 days) is not effective.^[2,19]^ Emerging research suggests that vaccines could potentially serve as trigger factors for certain inflammatory autoimmune disorders that affect the nervous system. This phenomenon might be linked to the immune system response to human papilloma virus (HPV) virus-like particles, which can stimulate the immune system via a process known as “bystander activation.”^[10,20]^ Additionally, the secretion of IL-6 and TNF-α by dendritic cells could provide immunomodulatory signals that contribute to inflammatory reactions within the nervous system.^[10,21]^ Overall, research has indicated that inflammatory diseases affecting the central nervous system are more frequently observed in young individuals, Isaacs syndrome, a relatively rare disease, may also have this tendency and may be associated with an immune response following vaccination.^[10]^ So, there is some overlap between this group and the population that receives HPV vaccination. Nonetheless, ISAACS syndrome symptoms in young patients vaccinated with HPV may indicate inflammatory activity affecting the peripheral nervous system. Our findings contribute to the expanding phenotypic spectrum of CASPR2 and LGI1 autoimmune syndromes, suggesting that these 2 antigens, particularly CASPR2, may potentially serve as novel target antigens and be implicated in the etiology of Isaac syndrome.

## 4. Conclusion

In summary, we have identified a unique case where a combination of LGI1 antibodies, CASPR2 antibodies, anti-phospholipid antibodies, anti-sulfate antibodies, TPO, ATG, and IgG E were present following the administration of the second dose of qHPV vaccine. This patient exhibited symptoms of Isaacs syndrome as well as Guillain-Barré-like features, expanding the phenotypic spectrum associated with CASPR2 and LGI1 autoimmune syndromes. Our findings suggest that Isaacs syndrome symptoms in young individuals who have received the HPV vaccine may involve autoimmune activity in the peripheral nervous system, potentially indicating a rare adverse event following HPV vaccination. The effectiveness of intravenous immunoglobulin therapy further supports this observation in patients. Although the exact pathogenesis of the disease related to the HPV vaccine remains unclear, further research is warranted to investigate this phenomenon.

## Acknowledgments

We thank the physicians who provided clinical support and clinical examination. We also wish to thank our patient and her family. We thank Professor Ren Haitao of Peking Union Medical College Hospital for his guidance in diagnosis.

## Author contributions

**Formal analysis:** BuFan Yang, Pei Xiao, Yu Jing.

**Investigation:** Yufeng Tang, Jingfeng Duan.

**Writing – original draft:** BuFan Yang.

**Writing – review & editing:** BuFan Yang, Wei Wei, Yufeng Tang.
